# Preparation and Physical Properties of Chitosan Benzoic Acid Derivatives Using a Phosphoryl Mixed Anhydride System

**DOI:** 10.3390/molecules17022231

**Published:** 2012-02-22

**Authors:** Duckhee Lee, Zhe Shan Quan, Chichong Lu, Jin Ah Jeong, Changhyun Song, Mi-Sun Song, Kyu Yun Chai

**Affiliations:** 1 The Division of Bio-Nanochemistry, The College of Natural Sciences, The Wonkwang University, Iksan City, Chonbuk, 570-749, Korea; Email: dl202@wonkwang.ac.kr (D.L.); chichonglu@hotmail.com (C.L.); mi_in_black@nate.com (J.A.J.); schh1004@cyworld.com (C.S.); misun4y@nate.com (M.-S.S.); 2 College of Pharmacy, Yanbian University, Yanji 133000, China; Email: quanzheshan@hanmail.net

**Keywords:** acylation, chitosan benzoate, benzoic acid, trifluoroacetic anhydride, phosphoric acid

## Abstract

Direct benzoylation of the two hydroxyl groups on chitosan was achieved using a phosphoryl mixed anhydride system, derived from trifluoroacetic anhydride (TFAA), benzoic acids (BAs), and phosphoric acid (PA). The reaction is operated as a one pot process under mild conditions that does not require neither an inert atmosphere nor dry solvents. The structures of the synthesized compounds were confirmed by NMR and IR spectroscopy. Solubility tests on the products revealed that they were soluble in organic solvents such as *N*,*N*-dimethylformamide (DMF), dimethylsulfoxide (DMSO), and acetone. In the meantime, a morphological study by scanning electron microscopy (SEM) evidently indicated that the chitosan benzoates underwent significant structural changes after the benzoylation.

## 1. Introduction

Chitin, the second abundant natural polymer in Nature, and chitosan, a partially deacetylated form of chitin, have recently received much of attention owing to their applicability in wide range of fields such as pharmaceuticals, cosmetics, agriculture, foods, and material sciences [1,2,3,4,5]. Their film and fiber-forming ability as well as biodegradability, biocompatibility, and abundance in Nature make them attractive materials in such application fields. However, although they have been excellent candidates for practical uses and commercialization, their uses in daily life or industry have been greatly limited due mainly to their low solubility in common solvents and poor processability. To impart better biological activity or improved physical properties like solubility in organic solvents and processibility on the polymer molecules derivatization is often performed. Modification by *O*-acylation in chitin and chitosan can be easily envisaged for such purposes. In the chitin case several methods using acid anhydride [6,7], mixed anhydride [8,9], acyl chlorides [10,11], benzoyl chloride and methanesulfonic acid [12], and *p*-toluenesulfonyl chloride, lithium chloride, and carboxylic acid [13] have been reported. Among them, the mixed anhydride method using trifluoroacetic anhydride, phosphoric acid, and carboxylic acid is most notable from the perspective of reaction yield, mild conditions, cheap price and recyclability of the reagents used [14,15,16]. This method has been already adopted by us for preparation of aliphatic and aromatic esters of chitin [17,18]. 

Meanwhile, in the case of chitosan, the situation is a little more complicated because there are two types of functional groups in the molecule, two hydroxyls at the 3,6-carbon position and an amino group at the 2-carbon position which is considered to be necessary for biological activities such as antibacterial or antiviral activity. Only a few acylations on chitosan are reported in the literature. Okamoto *et al*. used benzoyl chloride together with 4-dimethylaminopyridine functioning as a catalyst, to obtain tribenzoyl chitosan [19,20]. They also showed that *N*-(2-carboxy)benzoyl chitosan and *N*-phthaloyl chitosan derivatives could be synthesized using phthalic anhydride [21]. The acyl chloride (benzoyl) method was also employed by Feng *etc*. [22], Vasnev *etc*. [23] Zhu *etc*. [24], and Jiangtao [25]. The method is carried out either with acyl chloride alone or together with strong acids such as methanesulfonic acid playing role as a catalyst and simultaneously a protecting group for the free amino group. 

The carboxylic acid anhydride method was often used for *N*-acylation of chitosan [26,27,28,29,30]. And mixed anhydride method using trifluoroacetic anhydride and carboxylic acid was also successfully applied to the synthesis of acylated chitins by Yang [8] and acylated (aliphatic) chitosans by us, respectively [31]. This method can be carried out with or without phosphoric acid. The phosphoric acid-promoted method has several advantages such as a shorter reaction sequence (one step), gentle reaction conditions, low cost of the raw materials used, simplicity of operation in preparative process, and scalability, compared to other methods mentioned above. Besides, selective esterification on the hydroxyl group attached to the primary carbon atoms of 6-positions in the glucosamine unit of chitosan polymer can be directly achieved [31].

To the best of our knowledge, nevertheless, so far, there has been no report on studies using such a reaction system for preparing aromatic carboxylic acid derivatives of chitosan. In continuation of our research on acylated chitosans, we wish to report a new synthetic method using the above reaction system for preparing chitosan benzoyl esters and a study of the physical properties of the products. 

## 2. Results and Discussion

**Scheme 1:**
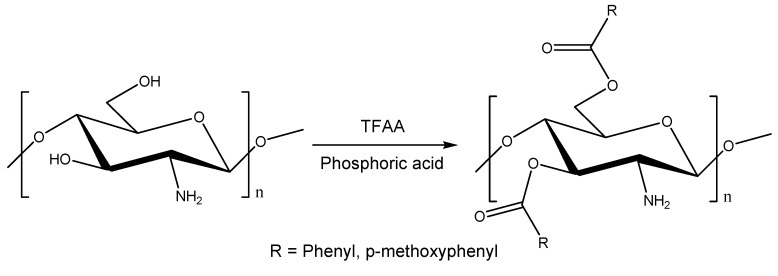
The benzoylation of chitosan using TFFA, benzoic acid, and phosphoric acid.

The benzoylation of chitosan (CTS) were carried out using the mixed anhydride derived from trifluoroacetic anhydride (TFFA), benzoic acids (BAs, benzoic acid and *p*-methoxybenzoic acid), and phosphoric acid (PA). This synthetic method was chosen because it was expected to require lower molar ratios of BAs and TFFA to chitosan than the reaction using mixed anhydride derived from TFFA and BAs (Scheme 1). Both of the reactions with benzoic acid and *p*-methoxybenzoic acid proceeded smoothly in the absence of solvent to give the chitosan benzoates in good yields, although they were more sluggish than the reactions with aliphatic carboxylic acids [31]. The yields of the reaction are shown below along with the reaction conditions (Table 1). 

**Table 1:** The yields of the chitosan benzoylation using TFFA, BAs, and PA.

Reactants	Chitosan (g)	TFAA (g)	Benzoic acid (g)	H_3_PO_4_ (g)	Product/Yield
* Molar ratio	1	8	2	2	
Input	1g	6.56	1.44	0.343	^a^ CTS-b/1.8 g
Input	1g	6.56	** 1.80	0.343	^b^ CTS-m/1.5 g

* based on the glucosamine unit, ** *p*-methoxybenzoic acid. ^a^ chitosan *p*-methoxybenzoate (CTS-m); ^b^ chitosan benzoate (CTS-b).

**Figure 1:**
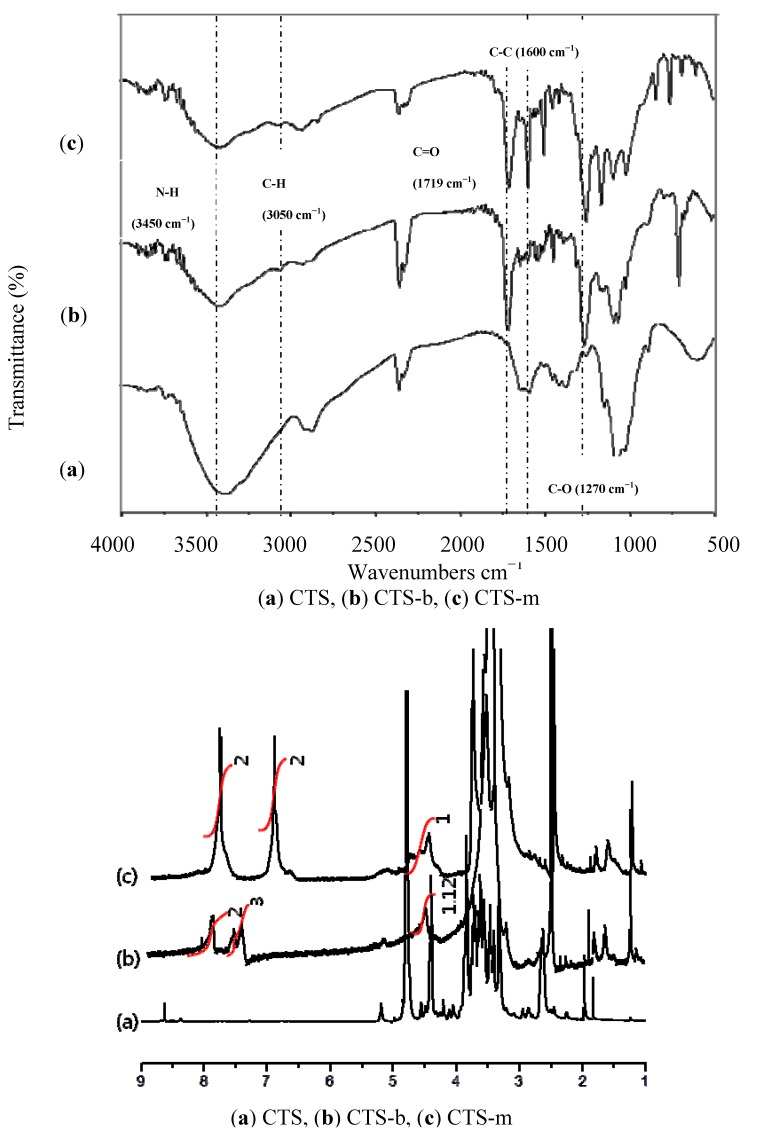
The IR and NMR spectra of the benzoyl chitosans and the parent chitosan.

Formation of a ester bond between BAs and chitosan in the products was confirmed using infrared and NMR spectroscopy. As shown in Figure 1, a new strong and broad peak around 1,735 cm^−1^ corresponding to the stretching absorption of the ester carbonyl, and another strong peak around 1,210 cm^−1^ corresponding to the stretching absorption of the ester C–O single bond were found in their FT-IR spectra, respectively. Both of the peaks are absent in the parent chitosan. Additional evidences for the structure of the products were obtained from the ^1^H-NMR spectra of the products shown in Figure 1, which was taken in DMSO-*d6* solvent with small amount of deuterated water. Characteristic signals for the protons attached to the aromatic carbon atoms and the anomeric carbon were observed between δ 7.5 and δ 7.9, and at δ 4.5, respectively. The degree of substitution was determined to be roughly 0.89 (benzoate) and 1.0 (methoxy benzoate) respectively by integral of the peaks.

The solubility of the products in organic solvents was tested using DMSO, DMF, acetone, ethanol, and THF. The results are shown in Table 2. Both of the synthesized benzoylated chitosan derivatives were soluble in DMSO, DMF, and acetone, but they were insoluble in THF and ethanol. 

**Table 2:** The solubility of the benzoyl chitosans in various organic solvents.

* Solubility of products					
Solvent	DMSO	DMF	EtOH	Acetone	THF
CTS-b	3.52 g/mL	3.44 g/mL	IS	1.54 g/mL	IS
CTS-m	3.30 g/mL	3.36 g/mL	IS	1.62 g/mL	IS

IS = insoluble. * Determined at r.t.

**Figure 2:**
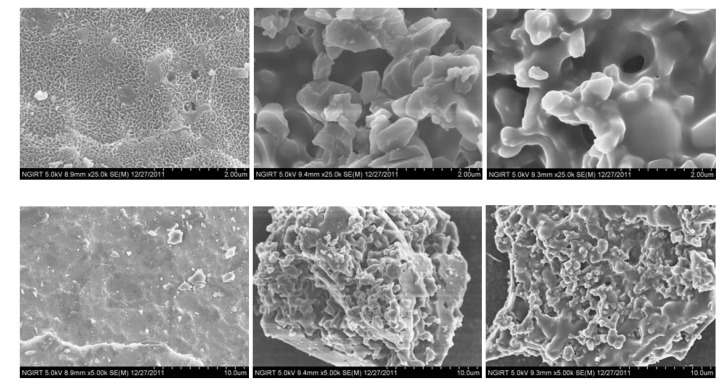
The SEM images of the parent chitosan and the synthesized benzoyl chitosans. The upper images: 25,000 times magnified images; The lower images: 5,000 times magnified images; Left: CTS; middle: CTS-b; right: CTS-m.

Morphological studies were carried out using a SEM. As shown in Figure 2, the SEM images of the surface of the parent chitosan and the synthesized benzoyl chitosans show clear differences between them. The numerous pores in the parent chitosan are irregularly arranged and the diameter of the pores are roughly 0.5–2.0 µm. The shape of the pores is also comparatively uniform. However, the pores in the benzoylated chitosans are various in shape and are also much bigger in size (5–20 µm). The magnified appearance of the parent chitosan looks like a piled fishing net, but that of benzoylated chitosans (benzoic acid and *p*-methoxybenzoyl chitosan) resembles slightly melted down coral-shaped dendritic structures. These images implies that the backbone of the chitosan benzoates-polymers undergoes significant structural changes after the acylation reaction, probably due to breaking of hydrogen bonds present in the parent chitosan and interaction between the newly introduced hydrophobic phenyl groups.

## 3. Experimental

### 3.1. General

The NMR spectra of the products were recorded at room temperature (20 °C) on a JEOL JNM-ECP FT-NMR spectrometer (500 MHz) using 5 mm diameter tubes. Samples were dissolved in DMSO-*d6* at the concentration of 15 mg/mL. The IR spectra were recorded on a Shimadzu Prestige-21 FT-IR spectrometer. The samples were prepared as a KBr pallet and scanned against a blank KBr pellet background at wave number ranging from 4,000 to 400 cm^−1^. The surface morphology of the parent chitosan and the products were observed by scanning electron microscopy (SEM), using a S-4800 instrument (Hitachi, Tokyo, Japan). The samples were analyzed at an accelerating voltage of 5 kV and 5,000–25,000× magnification. The solubility of the products was tested by dissolving a weighted sample in selected solvents at room temperature until the sample no longer became to be dissolved.

### 3.2. Materials

Chitosan (Mwt: below 1,000, degree of deacetylation: about 90%, moisture content: 6.8%) was purchased from the Kitto Lift (Pyeongtaek, Korea), TFAA (trifluoroacetic anhydride) from Acros Organics B.V.B.A (Geel, Antwerp, Belgium); benzoic acid and *p*-methoxybenzoic acid, 85% phosphoric acid from Sigma Aldrich (Yongin, Korea); dimethyl sulfoxide-*d6* from Cambridge Isotope Laboratories, Inc. (Andover, MA, USA). All other chemicals are of reagent grade. The materials were used as received without further purification.

### 3.3. Synthesis

The chitosan benzoate was synthesized as follows: benzoic acid (1.44 g) was added portionwise to a flask containg TFAA (6.56 g) at 40 °C over 1 h with stirring. The reaction mixture was heated to 50 °C and stirred for further 1 h until the reaction mixture became a pale yellow transparent liquid. After the reaction temperature was lowered to 20 °C and 85% phosphoric acid (0.343 g) was added all at once to the solution and stirred for 10 min. further, prior to addition of the chitosan (1.0 g, equivalent to 12.10 mmol of hydroxyl groups). The reaction mixture was stirred at 20 °C for 0.5 h further, heated up to 45–50 °C and vigorously stirred for 20 h at the same temperature until it became a brown syrup. Anhydrous ethyl alcohol (about 5 g) was added to the syrup and stirred for 10 min. After volatile materials were completely removed by a rotary evaporator under reduced pressure, the solution was well mixed with anhydrous ethyl alcohol (30 mL) and filtered through a gauze cloth. The filtrate was again concentrated under reduced pressure to a syrup. The products were precipitated by dissolving the syrup in a 1:1 (v/v) mixture of ethanol/acetone (total 10–15 mL) followed by addition of diethyl ether (300 mL) and kept at a refrigerater (0–5 °C) for 2–3 h. The product was obtained as solid powder after filtration and washing several times with diethyl ether (18 L). The dissolving and washing processes were repeated more than 20 times until no colour appeared. The powder was dried at 50 °C under high vacuum for 5 h.

^1^H-NMR: for the chitosan *p*-methoxybenzoate (DMSO-*d6* and 1 drop of D_2_O): δ 7.9 (doublet, aromatic Hs: *ortho*), δ 7.0 (aromatic Hs: *meta*), δ 4.5 (anomeric C–H, 1.0 H), δ 3.2–3.9 (C–Hs of carbohydrate backbone). Degree of substitution: 1.0.

For the chitosan benzoate (DMSO-*d6* and 1 drop of D_2_O): δ 7.95 (broad singlet, aromatic Hs: *meta*), δ 7.6 (broad singlet, aromatic Hs: *para*), δ 7.5 (broad singlet, aromatic Hs: *ortho*), δ 4.5 (anomeric C–H, 1.12 H), δ 3.2–3.9 (C–Hs of carbohydrate backbone). Degree of substitution: 0.89.

FT-IR: For the chitosan benzoate (KBr pellet): around 3,450 cm^−1^(N-H bonds), around 3,050 cm^−1^ (aromatic C–H bonds), 2,960 cm^−1^ (aliphatic C–H bonds), 1,710 cm^−1^ (aromatic ester carbonyl), 1,270 cm^−1^ (aromatic C-H bonds).

For the chitosan *p*-methoxybenzoate (KBr pellet): around 3,450 cm^−1^ (N–H bonds), around 3,050 cm^−1^ (aromatic C–H bonds), 2,960 cm^−1^ (aliphatic C–H bonds), 1,710 cm^−1^ (aromatic ester carbonyl), 1,270 cm^−1^ (aromatic C–H bonds).

## 4. Conclusions

A new synthetic method suitable for preparation of *O*-benzoyl chitosans, for not only academic small scale research, but also industrial mass production, has been developed using TFFA/H_3_PO_4_-mediated acylation. The synthesized chitosan derivatives were soluble in organic solvents such as DMF, DMSO, Acetone, but they were all insoluble in THF and ethanol. The morphological study perfomed by SEM on the parent chitosan and the synthesized benzoyl chitosans suggests that the parent chitosan undergoes significant structural change after the benzoylation reaction. These structural changee in the benzoyl chitosans can be interpreted as a result of breaking of the hydrogen bonds and the hydrophobic interaction between newly introduced phenyl groups. 

Thus, both of the synthetic method and the synthesized benzoyl chitosans, are expected to play an important role in the field of chitosan in future, especially for drug delivery, cosmetics, wound healing, and chromatographic separation technology, *etc*. 
